# Malignant peritoneal mesothelioma interactome with 417 novel protein–protein interactions

**DOI:** 10.1038/s44276-024-00062-w

**Published:** 2024-05-24

**Authors:** Kalyani B. Karunakaran, Madhavi K. Ganapathiraju

**Affiliations:** 1 Supercomputer Education and Research Centre, Indian Institute of Science, Bengaluru, 560012 India; 2 Department of Biomedical Informatics, School of Medicine, and Intelligent Systems Program, School of Computing and Information, University of Pittsburgh, 5607 Baum Blvd, 5th Floor, Pittsburgh, PA 15206 USA; 3 Carnegie Mellon University in Qatar, Doha, Qatar

## Abstract

**Background:**

Malignant peritoneal mesothelioma (MPeM) is an aggressive cancer affecting the abdominal peritoneal lining and intra-abdominal organs, with a median survival of ~2.5 years.

**Methods:**

We constructed the protein interactome of 59 MPeM-associated genes with previously known protein-protein interactions (PPIs) as well as novel PPIs predicted using our previously developed HiPPIP computational model and analysed it for transcriptomic and functional associations and for repurposable drugs.

**Results:**

The MPeM interactome had over 400 computationally predicted PPIs and 4700 known PPIs. Transcriptomic evidence validated 75.6% of the genes in the interactome and 65% of the novel interactors. Some genes had tissue-specific expression in extramedullary hematopoietic sites and the expression of some genes could be correlated with unfavourable prognoses in various cancers. 39 out of 152 drugs that target the proteins in the interactome were identified as potentially repurposable for MPeM, with 29 having evidence from prior clinical trials, animal models or cell lines for effectiveness against peritoneal and pleural mesothelioma and primary peritoneal cancer. Functional modules related to chromosomal segregation, transcriptional dysregulation, IL-6 production and hematopoiesis were identified from the interactome. The MPeM interactome overlapped significantly with the malignant pleural mesothelioma interactome, revealing shared molecular pathways.

**Conclusions:**

Our findings demonstrate the utility of the interactome in uncovering biological associations and in generating clinically translatable results.

## Introduction

Internal organs, such as the heart and lungs, and body cavities, such as the thoracic and abdominal cavities, are covered by a thin, slippery layer of cells called the “mesothelium”. Mesothelioma is a rare but highly aggressive cancer that originates from this lining, constituting the subtypes pericardial (heart), pleural (lung) and peritoneal (abdomen) mesothelioma; it is usually found in advanced stages and has a median survival of one year [1]. Mesothelioma is intricately linked with exposure to asbestos but with a long latency period of a few decades between exposure and the occurrence of the disease. It does not have a non-invasive pre-malignant phase, unlike other cancers. The focus of this work is on the genetics and biological mechanisms of malignant peritoneal mesothelioma (MPeM).

The peritoneum is a serosal membrane made up of two layers of mesothelial cells lining the abdominal cavity and intra-abdominal organs. MPeM affects this peritoneal lining and grows within the peritoneal space [1]. Patients may exhibit symptoms such as weight loss, shortness of breath, chest and abdominal pain, increased abdominal girth and peritoneal effusion between the ages of 40–65 years [1]. MPeM constitutes a substantial percentage (15–20%) of all mesothelioma diagnoses and is distinct from malignant pleural mesothelioma (MPM) due to its limited association with asbestos exposure (8% compared to 80% for MPM) [2]. MPeM was more apparent among patients with a history of abdominal surgeries rather than asbestos exposure [1, 2]. Peritoneal cases are also becoming increasingly prevalent among mesothelioma patients without occupational exposure, given the current scenario in which the population of asbestos-exposed individuals is diminishing [1]. MPeM exhibits a highly variable pattern of disease progression, and patients often develop the risk of postoperative morbidity and mortality [1]. MPeM has a higher median survival rate than pleural mesothelioma (31 months versus 14 months) [3] and is currently treated with a combination of pemetrexed and cisplatin [1]. Given the unique features of MPeM and its fatal nature, it is imperative that the molecular mechanisms underlying this disease are expeditiously discovered.

Factors predisposing patients to MPeM remain unclear [2]. However, MPeM is known to be proportionally more prevalent than MPM among patients with germline mutations and without a history of asbestos exposure (25% versus 7% [4]) [1]. Multiple studies have examined the genetic underpinnings of MPeM (see Table 1). Altogether, these studies reported 59 MPeM-associated genes that harboured mutations, copy number aberrations, and rearrangements or whose expression correlated with poor prognosis in MPeM patients, and reduced cell survival or unfavourable drug responses in MPeM surgical specimens [5–13]. The Cancer Genome Atlas (TCGA) also provided comprehensive genomic datasets of mesothelioma, describing mutations in BAP1, CDKN2A, LATS1, LATS2, MSH2, NF2, PBRM1, PTCH1, RBFOX1, SETD2, SETDB1 and TP53 (included in Table 1) [14]. The next step to discovering biological mechanisms is to understand how these genes play a role in the disease. To address this, we constructed the protein-protein interaction network (or the ‘interactome’) of these MPeM-associated genes, including hitherto unknown interactions that are computationally predicted and are considered with high-confidence to be true interactions. Further, using various bioinformatics methods, we gained insights into the biological processes underlying MPeM, and identified repurposable drugs.

**Table 1:** Publications examining the genetic basis of MPeM, related details, and the gene lists from each used in our study.

Study	Study methods	Patient samples	MPeM-associated genes
Hung et al. [13].	Immunohistochemistry, fluorescence in situ hybridisation (FISH), targeted next-generation sequencing of tumour DNA and RNA	88 consecutive patients with peritoneal mesotheliomas diagnosed at a single institution between 2005 and 2015	ALK
Joseph et al. [5].	Next-generation sequencing 510 cancer-related genes, immunohistochemistry	13 patients with malignant mesothelioma arising in the peritoneal cavity	ARID1A, BAP1, DDX3X, NF2, SETD2, TERT, WT1
Ugurluer et al. [6].	Next-generation sequencing testing, descriptive and Kaplan-Meier statistics	11 patients with somatic cancer-related mutations	AR, ASXL1, BAP1, BRIP1, CDK12, DAXX, EPHB1, ESR1, FGF6, IRS2, JAK1, KDM6A, KDR, KEAP1, KMT2A, MET, MRE11, MTOR, NSD1, SETD2, TSC1
Chirac et al. [7].	Comparative genomic hybridisation using the Agilent Human Genome CGH 180 K array	MPeM samples from 33 patients	ADAM3A, ARHGAP22, BAP1, CDH5, CDKN2A, CHEK2, CTNNB1, DPYD, EGFR, HRAS, IGKC, JUN, MAPK8, NF2, NR2F2, PTEN, RASSF1, RB1, RHEB, RICTOR, SDHB, SMARCB1, STK11, TRIO, VEGFB
Foster et al. [8].	Evaluation of patient tumours for mutations in the catalytic TK-domain, treatment of patients with cytoreductive surgery, COS-7 cell expression model to determine mutation activating profiles and response to erlotinib	MPeM tumours from 29 patients, 25 of whom were treated with cytoreductive surgery	EGFR
Hung et al. [9].	Targeted next-generation sequencing, immunohistochemistry	Diffuse peritoneal mesotheliomas from 26 patients	ARID1B, BAP1, CDKN2A, CHEK2, NF2, PBRM1, PRDM1, SETD2, SUZ12, TP53, TRAF7
Pillai et al. [10].	Immunohistochemistry, prognostic significance using the Kaplan-Meier method	MPeM samples from 42 patients	MUC1
Varghese et al. [11].	Gene expression analyses, pathway-specific inhibition	Fresh pre-treatment MPeM tumour samples collected from 41 patients who underwent surgical cytoreduction and received regional intraoperative chemotherapy perfusion	PIK3CA, RICTOR
Zaffaroni et al. [12].	Immunohistochemistry	32 MPeM surgical specimens	BIRC5

Protein-protein interactions (PPIs) drive the biological processes in cells including signal transduction, formation of cellular structures and enzymatic complexes. The molecular mechanisms of disease are often revealed by the PPIs of disease-associated genes. For example, the involvement of transcriptional deregulation in pleural mesothelioma pathogenesis was identified through mutations detected in BAP1 and its interactions with proteins such as HCF1, ASXL1, ASXL2, ANKRD1, FOXK1 and FOXK2 [15]. PPI of BAP1 with BRCA1 was central to understanding the role of BAP1 in growth-control pathways and cancer; BAP1 was suggested to play a role in BRCA1 stabilisation [16, 17]. Studies on BAP1 and BRCA1 later led to clinical trials of the drug vinorelbine as a second-line therapy for MPM patients, and the drug was shown to have rare or moderate effects in MPM patients [18, 19].

Despite their crucial role in understanding disease mechanisms and discovering drugs, ~75% of estimated PPIs are unknown, and several disease-associated genes lack known PPIs. The human interactome may contain more than 600,000 PPIs [20], but only ~150,000 PPIs are known from PPI repositories such as HPRD [21] and BioGRID [22]. Experimental detection of PPIs using techniques such as co-immunoprecipitation (Co-IP) [23, 24] is time-consuming at large scale. Although systematic high throughput studies with yeast two-hybrid (Y2H) system [25] and affinity purification–mass spectrometry (AP–MS) [26] have helped discover tens of thousands of PPIs, a large part of the interactome remains unknown. We developed **HiPPIP** (high-precision protein–protein interaction prediction), a computational model deemed highly accurate by computational evaluations and experimental validations of 18 predicted PPIs, where all the tested pairs were shown to be true PPIs [27, 28].

We derived valuable insights from the analysis of disease-specific protein interactomes that included PPIs predicted by HiPPIP. Notably, we identified 2,156 novel PPIs for diseases such as MPM [29], schizophrenia [27], rheumatoid arthritis [30], and congenital heart disease [31, 32]. Our previous study that demonstrated the functional links of MPM-associated genes collected from various high throughput investigations within the MPM interactome, underscored the importance of interactome analysis in understanding the molecular basis of mesothelioma [29]. More than 85% of the genes in the interactome were supported by MPM-related genetic variant, transcriptomic and proteomic evidence. Furthermore, we experimentally validated 5 novel PPIs of MPM-associated genes and identified 5 repurposable drugs targeting the interactome proteins. This collective evidence motivated us to extend our interactome-based approach to the exploration of the genetic basis of MPeM.

In this work, we constructed the ‘MPeM interactome’ by assembling the known and computationally predicted PPIs of the genes associated with MPeM. Analysing this interactome within the context of peritoneal mesothelioma transcriptomic data, gene tissue specificity, prognostic relevance of genes in other cancers, and interconnections to MPM, we expanded our understanding of MPeM. We then investigated the pathways and functional modules associated with the interactome. Finally, we integrated drugs sourced from the Drug Bank repository [33] targeting at least one of the interactome proteins and performed comparative transcriptome analysis of drug-induced and MPeM-associated profiles to identify 29 repurposable drugs for MPeM.

## Results

**Fig. 1:**
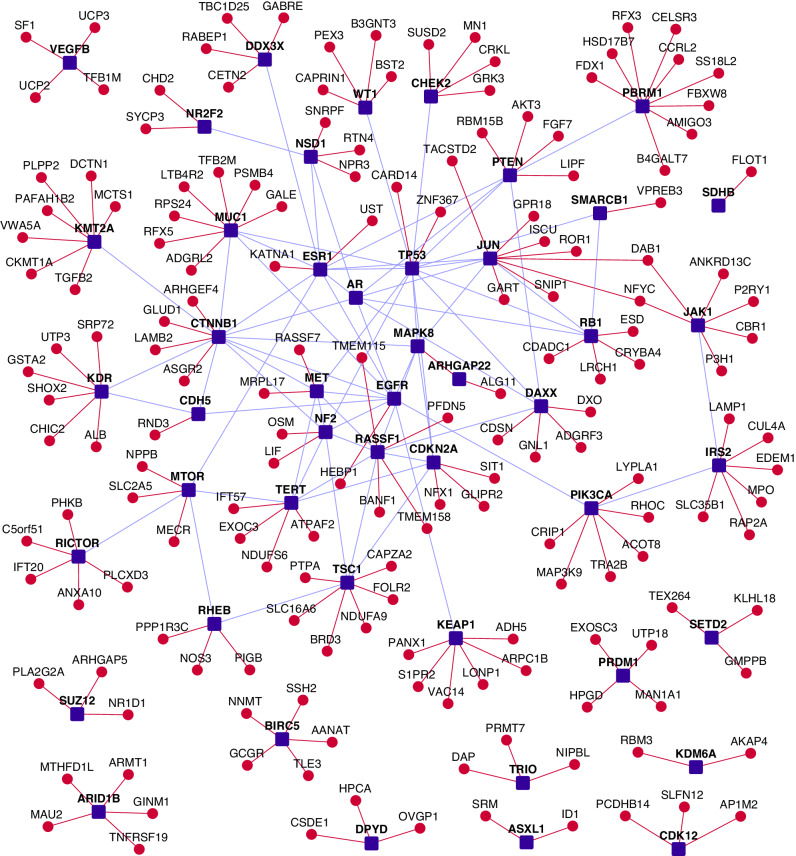
A partial network view of protein–protein interactions (PPIs) in the malignant peritoneal mesothelioma (MPeM) interactome: genes are shown as nodes and PPIs as edges. As the complete MPeM network is very large, only a partial view showing a large connected component of MPeM candidate genes and their novel interactors, all of which have MPeM-related transcriptomic evidence (Supplementary Data File 4), is shown. Legend: dark blue square-shaped nodes: MPeM candidate genes; red nodes/edges: novel interactors/interactions; light blue edges: known interactions.

PPIs of the MPeM-associated genes (or ‘core’ genes) shown in Table 1 were collected from HPRD [21] (Human Protein Reference Database) and BioGRID [22] (Biological General Repository for Interaction Datasets); see Supplementary Data File 1 for the reported gene alterations. The HiPPIP algorithm described in our earlier work was applied to MPeM genes to discover hitherto unknown PPIs [34]. HiPPIP computes features of protein pairs such as cellular localisation, molecular function, biological process membership, genomic location of the gene, and gene expression in microarray experiments and classifies the pairwise features as *interacting* or *non-interacting* based on a random forest model [27]. The ‘MPeM interactome’ assembled in this manner contained 4747 known PPIs and 417 novel PPIs connecting 58 MPeM-associated genes to 2747 known interactors and 306 novel interactors (Fig. 1 and Table 2, and for computer processing, also made available in Supplementary Data File 2). The 59th MPeM-associated gene ADAM3A had neither known nor novel PPIs.

**Table 2:** Novel PPIs of MPeM-associated genes. Number of known PPIs (‘K’) and novel PPIs (‘N’).

Gene	K	N	Novel Interactors
ADAM3A	0	0	
ALK	16	12	BIRC6, CLNS1A, DLG2, DNMT3A, HADHB, HMGB1, MSH2, NLRC4, RASA1, SMC1A, TSPAN16, TTC19
AR	265	0	
ARHGAP22	7	5	ALG11, GPRIN2, MAPK8, PTPN20, ZNF488
ARID1A	17	7	CDC123, EDEM1, GMEB1, SMPDL3B, TAF12, THEMIS2, VPS41
ARID1B	7	8	ARMT1, GINM1, MAU2, MTHFD1L, NCOA6, PLEKHG1, TNFRSF19, ZBTB2
ASXL1	6	9	DEFB115, DEFB116, FASN, ID1, IRAG1, MRPS7, NCOA6, POLD1, SRM
BAP1	27	2	PARP3, PLN
BIRC5	25	8	AANAT, AKAP6, CSNK1D, FASN, GCGR, NNMT, SSH2, TLE3
BRIP1	10	4	HPN, MRPS23, PATZ1, PECAM1
CDH5	21	7	CA7, CDH1, CDH16, CDH3, NQO1, PYGB, RND3
CDK12	9	9	AP1M2, CDKN1A, FNDC8, GSDMB, PCDHB14, PCNT, PDLIM7, RPL13, SLFN12
CDKN2A	168	5	CA9, DNAI1, GLIPR2, NFX1, SIT1
CHEK2	80	5	CRKL, FUS, GRK3, MN1, SUSD2
CTNNB1	187	11	ARHGEF4, ASGR2, CCR1, CDC25A, CDK5, GLUD1, GNAI2, LAMB2, PTPRB, SSTR3, TJP1
DAXX	113	10	ADGRF3, CDSN, COL11A2, DXO, FBXO25, GNL1, GTF2H4, H2BC15, IKBKE, NRG2
DDX3X	92	4	CETN2, GABRE, RABEP1, TBC1D25
DPYD	3	8	CPB2, CSDE1, HPCA, KCNJ4, OVGP1, RPS6KA3, SRC, SULT2A1
EGFR	409	4	HEBP1, PHKG1, POM121L12, STAG3L4
EPHB1	13	10	AADAC, ACP3, AMOTL2, CTPS1, GM2A, GTF2E1, MBD4, MRPL3, PCCB, UQCRC2
ESR1	365	6	DDX43, FNDC1, KATNA1, RPL27A, SPDEF, UST
FGF6	5	2	CELSR1, KLRC2
HRAS	146	7	C6orf62, CCL4, HBG1, IGF2, INS, KCNQ1, ZFP36L2
IGKC	4	2	PLGLB2, REG3A
IRS2	40	10	CDKL1, CUL4A, EDEM1, LAMP1, MPO, NRAS, PROZ, PTPRR, RAP2A, SLC35B1
JAK1	72	8	ANKRD13C, CBR1, DAB1, DHX9, NFYC, P2RY1, P3H1, VNN2
JUN	180	9	DAB1, GART, GPR18, ISCU, NFYC, PML, ROR1, SNIP1, TACSTD2
KDM6A	11	7	AKAP4, BMP15, ELK1, HSD17B10, MAGED2, RBM3, ZNF157
KDR	60	8	ALB, CACNA1S, CHIC2, GSTA2, KIT, SHOX2, SRP72, UTP3
KEAP1	77	15	ADH5, ARPC1B, BNIP3L, CARM1, ERBIN, LONP1, PANX1, RTN4R, S1PR2, SDC1, SENP1, SLC5A5, VAC14, ZNF177, ZNF266
KMT2A	31	13	CKMT1A, DCTN1, EXOSC5, IL10RA, INPPL1, LAYN, MCTS1, PAFAH1B2, PLAAT4, PLPP2, RDX, TGFB2, VWA5A
MAPK8	137	8	ANXA8L1, ARHGAP22, CDC42, GDF10, GPRIN2, MT-CO1, PTPN20, TIMM23B
MET	112	12	CAMSAP3, CAV1, FOXA3, KCND2, KLK2, MRPL17, PABPC1, RASSF7, SH3KBP1, SLC26A3, SLC26A4, SND1-IT1
MRE11	20	8	-, CXCR5, DYNC1H1, ENDOD1, GPR83, JRKL, MLF2, TMEM126A
MTOR	52	7	CA6, MECR, NPPB, PIK3CD, SLC2A5, SLC45A1, SLC9A1
MUC1	134	12	ADGRL2, E2F2, GALE, IKBKE, LTB4R2, OAZ1, PKLR, PSMB4, RFX5, RPS24, SYT13, TFB2M
NF2	74	4	DRG1, LIF, OSM, PCNA
NR2F2	18	3	CHD2, RLBP1, SYCP3
NSD1	36	5	NPR3, PRKCD, RING1, RTN4, SNRPF
PBRM1	11	11	AMIGO3, B4GALT7, CCRL2, CELSR3, FBXW8, FDX1, HSD17B7, PCBP4, RFX3, SS18L2, TMIE
PIK3CA	70	8	ACOT8, ALCAM, CRIP1, LYPLA1, MAP3K9, PRKCI, RHOC, TRA2B
PRDM1	10	6	EXOSC3, FRK, HPGD, MAD2L1BP, MAN1A1, UTP18
PTEN	356	9	AKT3, ARL3, COL18A1, FGF7, KIF20B, LIPF, NCSTN, NR3C1, RBM15B
RASSF1	70	5	BANF1, LARS2, PFDN5, TMEM115, TMEM158
RB1	190	9	CDADC1, CNTN3, COX17, CRYBA4, CSK, ESD, LRCH1, MTRF1, PCDHB5
RHEB	29	6	CENPE, MAPK15, NOS3, PIGB, PPP1R3C, PTPRN2
RICTOR	11	8	ANXA10, C5orf51, CTSW, IFT20, MROH2B, PHKB, PLCXD3, SIAH1
SDHB	6	9	CA6, FLOT1, MFAP2, RAP1GAP, RPL11, SLC45A1, SLC9A1, STMN1, TARBP1
SETD2	8	6	EXOSC7, GMPPB, KLHL18, NDUFAF3, TEX264, TMA7
SMARCB1	112	3	CNTNAP3, MYO18B, VPREB3
STK11	134	5	FAM98B, MADCAM1, PPP6R1, SH3GL1, ZNF195
SUZ12	17	9	ARHGAP5, CDK5R1, KLRC1, NOL4, NR1D1, PLA2G2A, RBBP4, SLC2A2, TBC1D29P
TERT	80	9	ATPAF2, EXOC3, HMGB2, ICE1, IFT57, IRS1, NDUFS6, PDCD6, PTMA
TP53	489	6	CARD14, CFHR3, MMP10, POLR2A, RCVRN, ZNF367
TRAF7	12	6	BTBD1, HAGHL, HDGF, NUDT16L1, PBXIP1, PPIC
TRIO	13	5	DAP, DNAH5, MARCHF6, NIPBL, PRMT7
TSC1	99	11	BRD3, CAPZA2, FCN1, FOLR2, NDUFA9, PAEP, PTPA, SLC16A6, TRAF2, TUBB4B, ZNF79
VEGFB	6	5	FOSL1, SF1, TFB1M, UCP2, UCP3
WT1	64	8	B3GNT3, BST2, CALML5, CAPRIN1, FJX1, HIPK3, PAX6, PEX3

**Fig. 2:**
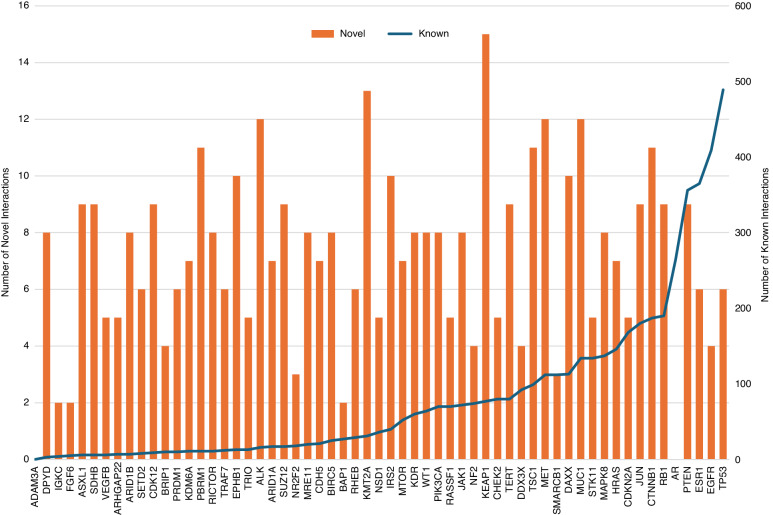
Number of protein-protein interactions: the MPeM-associated genes are listed along the *x*-axis, arranged in the ascending order of their number of known protein–protien interactions. The number of novel predicted PPIs and previously known PPIs are shown as red bars on the primary axis (left) and blue lines on the secondary axis (right). For example, DPYD has three known PPIs and 8 novel PPIs, and AR has 265 known and zero novel PPIs.

The number of known and computationally predicted novel PPIs for each of the MPeM genes are shown in Fig. 2 and Table 2; the novel interactors are also listed in Supplementary Data File 3. Thirteen genes had 10 or less interactions each and 73 novel PPIs were predicted for all of the genes combined. There are 21 hub genes that had more than 75 known PPIs each and 160 novel PPIs were predicted for all of the genes combined.

### Overlap of the MPeM interactome with transcriptomic data

198 out of 306 (65%) novel interactors and 2353 (75.6%) proteins overall of the MPeM interactome showed differential gene expression in pre-clinical models and human tumour specimens of peritoneal mesothelioma (see Table 3 and Supplementary Data File 4). These included human orthologues of genes differentially expressed in MPeM tumour specimens from patients, peritoneal mesotheliomas of rats, either spontaneously or chemically induced, mouse peritoneal mesothelioma cell lines resulting from crocidolite asbestos, and human peritoneal mesothelial lines exposed to crocidolite. These overlap studies confirmed the biological validity of the MPeM interactome by demonstrating its relevance in the context of rodent xenograft/cell line mesothelioma models and human mesothelial cell lines.

**Table 3:** Transcriptomic datasets enriched in the MPeM interactome, with the number of differentially expressed genes (DEGs) and *p*-value and odds ratio of enrichment.

Transcriptomic dataset (with reference to source publication)	Number of DEGs in the interactome	*P*-value of enrichment	Odds ratio of enrichment
Granulocytic myeloid-derived suppressor cells (G-MDSCs) from spleens of mice bearing AB12 mesothelioma grafts versus naive neutrophils (GSE43254 [56])	975	2.02E−14	1.21
Neutrophils infiltrating AB12 mesothelioma tumour grafts versus naive bone marrow-derived neutrophils (GSE43254 [56])	1006	1.97E−17	1.24
BCA-induced peritoneal mesothelioma versus non-transformed mesothelial cell line	533	1.08E−04	1.15
O-Nitrotoluene (O-NT) induced peritoneal mesothelioma versus non-transformed mesothelial cell line (GSE4682 [57])*	332	–	–
Spontaneous malignant mesotheliomas from 2-year-old rats versus normal mesothelial Fred-PE cells (GSE47581 [58])*	794	–	–
LP9 mesothelial cells exposed for 8 h to 5 µg/cm^2^ *crocidolite asbestos* versus untreated mesothelial cells (GSE14034 [82])	303	6.39E−08	1.32
LP9 mesothelial cells exposed for 8 h to 5 µg/cm^2^ *crocidolite asbestos* versus untreated mesothelial cells (GSE63966 [83])	560	1.24E−05	1.16
LP9 mesothelial cells exposed for 8 h to 1 µg/cm^2^ *crocidolite asbestos* (GSE14034 [82])	85	8.76E−04	1.38
Primary peritoneal mesothelial HM3 cells exposed to 5 µg/cm^2^ *crocidolite asbestos* for 8 h (GSE63966 [83])	797	3.99E−12	1.22
Biphasic versus epithelial peritoneal mesothelioma tumour specimens [35]	118	2.17E−19	2.25
Lungs of mice exposed to crocidolite fibres [84]	322	3.5E−13	1.44
Lungs of mice exposed to wollastonite fibres [84]	23	0.044	1.43

Note: (*) A considerable number of genes in the interactome were differentially expressed in rat models of spontaneous and induced peritoneal mesothelioma, but their overlaps were not statistically significant.

In order to examine whether the interactome showed preferential enrichment for any specific subtype of peritoneal mesothelioma, we computed its overlap with genes differentially expressed in biphasic versus epithelial peritoneal mesothelioma tumour specimens and vice versa [35]. Significant enrichment was found with biphasic mesothelioma but not with epithelial mesothelioma (Table 3 and Supplementary Data File 4). This overlap included 4 genes predicted as novel interactors of 4 MPeM-associated genes (MPeM genes are shown in bold): **ARID1A**-TAF12, **PIK3CA**-LYPLA1, **EPHB1**-MRPL3 and **KEAP1**-LONP1. Hence, the interactome with over 100 genes specific to the biphasic subtype of MPeM will prove valuable for investigating this relatively rarer subtype compared to the epithelioid and sarcomatoid subtypes [36].

Diffuse MPeM is known to share similar clinical presentation, morphology and immunostaining profiles with ovarian/primary peritoneal serous carcinoma (OC/PPC), and may hence be indistinguishable from the latter [37]. Gene expression signatures characterising these two tumours have been identified in an attempt to elucidate the molecular differences distinguishing them from one another [37]. We computed the overlap of the MPeM interactome with these expression profiles (see Supplementary Data File 4**)**. Out of the 12 genes in the interactome found to be differentially expressed in OC/PPC versus diffuse MPeM (including the MPeM-associated gene ESR1), 3 were predicted as novel interactors of MPeM-associated genes: **HRAS**-IGF2, **JUN**-TACSTD2, and **CHEK2**-SUSD2. Eight genes, including the MPeM-associated gene, KDR were found to be differentially expressed in diffuse MPeM versus OC/PPC. This analysis helped pinpoint the genes that distinguish MPeM from other morphologically and histogenetically similar tumours.

In summary, these overlap studies validated the relevance of MPeM interactome to MPeM tumours in rodent models and human patients, identified genes specific to MPeM subtypes and those aiding in differential diagnosis from other cancers. The interactome can be used as a mechanistic framework for investigating MPeM-related genes.

### Tissue-specificity of the genes in the MPeM interactome

**Fig. 3:**
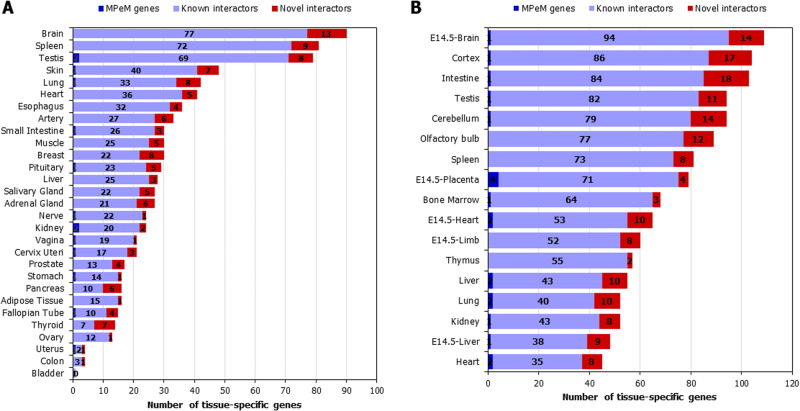
**A** Tissue-specificity of malignant peritoneal mesothelioma (MPeM) interactome genes in human organs: Tissue-specific expression of the genes in the interactome was examined using GTEx data. The graph shows the number of genes from the interactome that exhibit tissue specificity. The genes show at least 5-fold higher expression in a tissue (‘tissue-enriched’) or a group of 2-7 tissues compared to all the other tissues (‘group-enriched’). **B** Tissue-specificity of malignant peritoneal mesothelioma (MPeM) interactome genes in mouse organs: Tissue-specific expression of the genes in the interactome was examined using mouse ENCODE data. The graph shows the number of genes from the interactome that exhibit tissue specificity. The genes show at least 5-fold higher expression in a tissue (‘tissue-enriched’) or a group of 2–7 tissues compared to all the other tissues (‘group-enriched’).

We studied tissue-specific expression of the interactome genes using mouse ENCODE and GTEx data [38, 39]. Genes with an expression >1 TPM (transcripts per million) and 5-folds higher in a single tissue (tissue-enriched) or 2–7 tissues (group-enriched) were included [40]. Unexpectedly, the top enriched organs were spleen and small intestine, and not abdominal organs lined by the peritoneum (Fig. 3A). The other human organs that shared many genes with the interactome were brain, testis, skin, lung, heart, oesophagus, artery and muscle (Fig. 3A). Similar trends were observed with mouse expression data, with the intestine, cortex, cerebellum, olfactory bulb, testis and bone marrow, and embryonic tissues such as E14.5 brain, E14.5 placenta and E14.5 heart, showing enrichment in the interactome (Fig. 3B). The interactome exhibited notable enrichment in human orthologues of mouse genes specific to spleen (81 genes, *P*-value = 0.019, odds ratio = 1.39) and thymus (57 genes, *P*-value = 0.028, odds ratio = 1.42) (Fig. 3B). Ten MPeM-associated genes had novel PPIs with the orthologues of 10 spleen-specific mouse genes, namely, **SMARCB1**-VPREB3, **JAK1**-VNN2, **RHEB**-NOS3, **ALK**-NLRC4, **IRS2**-MPO, **TSC1**-FCN1, **RICTOR**-CTSW, **HRAS**-CCL4 and **BIRC5**-AANAT (i.e. 10 novel interactors had spleen-specificity; MPeM genes are shown in bold).

We used BaseSpace Correlation Engine [41, 42] to identify human peritoneum-specific genes from the interactome. A gene was deemed specific to the peritoneum if its expression decrease in other tissues compared to the tissue of interest (i.e. specificity index) was > 0.8. Based on this, only 6 genes showed peritoneum-specific expression. OVGP1, a predicted interactor of the MPeM-associated gene DPYD, had moderate peritoneal specificity (specificity index = 0.57).

Altogether, the analysis of tissue-specific expression in the interactome revealed scarce peritoneum-specific expression and unexpected enrichment in lymphatic organs such as the spleen and thymus.

### Functional modules and pathways enriched in the MPeM interactome

We used the HumanBase toolkit [43] (https://hb.flatironinstitute.org/) to identify functional modules in the MPeM interactome. HumanBase employs shared k-nearest-neighbours and the Louvain community-finding algorithm to cluster the genes sharing the same network neighbourhoods and similar Gene Ontology (GO) biological processes into functional modules. Fourteen modules were detected, of which 11 had more than 4 proteins each (Table 4).

**Table 4:** Functional modules in the MPeM Interactome (with FDR-corrected *p*-value).

Module	Enriched GO biological process	FDR-corrected *p*-value
M1	Chromosome segregation	<1E−08
M2	Translation	<1E−08
M3	Hematopoiesis	<1E−08
M4	Covalent chromatin modification	<1E−08
M5	Transmembrane receptor protein tyrosine kinase pathway	<1E−08
M6	Histone modification	<1E−08
M7	mRNA metabolic process	<1E−08
M8	Cell–cell adhesion	2.86E−05
M9	Transmembrane receptor protein tyrosine kinase pathway	2.82E−04
M10	Transmembrane receptor protein tyrosine kinase pathway	2.64E−03
M11	Negative regulation of intracellular signal transduction	3.98E−03
M12	Negative regulation of hydrolase activity	5.84E−03
M13	Cell–cell junction assembly	5.97E−03
M14	Positive regulation of interleukin-6 production	0.023

Next, we identified the REACTOME pathways enriched in the MPeM interactome using the gene set analysis toolkit called WebGestalt (Supplementary Data File 5) [44]. WebGestalt computes the statistical significance of the association of the genes with a specific functional group (e.g. a Reactome Pathway) using Fischer’s exact test and Benjamini-Hochberg method for multiple test adjustment. The top-10 pathways associated with the MPeM are shown in Table 5 [45].

**Table 5:** Selected pathways associated with MPeM interactome (FDR-corrected *p*-value < 1E-15).

Pathway	Number of genes	MPeM genes	Novel interactors
Immune System	578	BIRC5, CTNNB1, DDX3X, HRAS, IRS2, JAK1, JUN, KEAP1, MAPK8, MRE11, MTOR, MUC1, NF2, PIK3CA, PTEN, RICTOR, TP53, TRAF7	AKT3, AP1M2, ARPC1B, BST2, BTBD1, CALML5, CAPZA2, CCL4, CENPE, CFHR3, CPB2, CRKL, DCTN1, DEFB115, DEFB116, FBXW8, FCN1, GM2A, GSTA2, KLRC1, KLRC2, LAMP1, LIF, MADCAM1, MPO, NCSTN, NLRC4, NOS3, OSM, PAFAH1B2, PANX1, PLA2G2A, PSMB4, PTPRN2, PYGB, RAP1GAP, REG3A, SIAH1, SLC2A5
Gene expression (Transcription)	517	AR, ARID1A, ARID1B, BIRC5, BRIP1, CDK12, CDKN2A, CHEK2, CTNNB1, DAXX, EGFR, ESR1, JUN, KMT2A, MET, MRE11, MTOR, PBRM1, PRDM1, PTEN, RB1, RHEB, RICTOR, SMARCB1, STK11, SUZ12, TP53, TSC1	AKT3, DNMT3A, GTF2E1, ICE1, KIT, NLRC4, NR1D1, PCBP4, PSMB4, SNRPF, TAF12, TFB2M, ZNF157, ZNF195, ZNF266, ZNF79
Developmental Biology	361	CTNNB1, EGFR, EPHB1, HRAS, IRS2, JUN, KDM6A, MAPK8, MET, NR2F2, PIK3CA, SUZ12, TRIO	AKT3, ARPC1B, CACNA1S, CDSN, DAB1, FOXA3, NCSTN, PKLR, PSMB4, RAP1GAP, RHOC, RPS24, SIAH1, SLC2A2
Cytokine Signalling in Immune system	269	BIRC5, IRS2, JAK1, JUN, MAPK8, MUC1, PIK3CA, TP53	BST2, CCL4, CRKL, GSTA2, LIF, OSM, PSMB4
Cell Cycle	247	BIRC5, BRIP1, CDKN2A, CHEK2, MRE11, RB1, TERT, TP53	AKT3, BANF1, CENPE, CETN2, DCTN1, MAU2, NIPBL, PCBP4, PCNT, POLD1, PSMB4, SYCP3
Cellular responses to stress	170	AR, CDKN2A, JUN, MAPK8, MRE11, MTOR, RB1, SUZ12, TP53	CAPZA2, DCTN1, ID1, PSMB4
DNA Repair	133	BAP1, BRIP1, CHEK2, MAPK8, MRE11, TP53	CETN2, CUL4A, MBD4, POLD1
Deubiquitination	125	AR, ASXL1, BAP1, ESR1, KEAP1, PTEN, TP53	PSMB4
MAPK family signalling cascades	153	EGFR, FGF6, HRAS, IRS2, JAK1, JUN, MET	DLG2, FGF7, KIT, NRG2, PSMB4
SUMOylation	104	AR, BIRC5, CDKN2A, DAXX, ESR1, SUZ12, TP53	CETN2, DNMT3A, SENP1

The identified modules and pathways could contribute to peritoneal mesothelioma development and progression (see Discussion), including dysregulated chromosome segregation, covalent chromatin modification, altered mRNA metabolic processes, disrupted translation, post-translational events, activation of transmembrane receptor protein tyrosine kinase pathways, disrupted cell–cell junction assembly, and cytokine signalling, particularly interleukin-6 production. The identification of hematopoiesis as an enriched module aligns with the enrichment of genes specific to the extramedullary hematopoietic sites, spleen and thymus, in the interactome (Fig. 3).

### Association with other cancers

The prolonged survival of carriers of MPeM-associated mutations (e.g. in BAP1 and TP53) has been linked to the occurrence of other cancers [1]. This connection between cancer prognosis and comorbidities in MPeM patients prompted us to explore the presence of prognostic genes from various cancers within the MPeM interactome. We systematically examined the overlap between the MPeM interactome and prognostic genes from 20 cancer types, using data from Pathology Atlas for gene expression and patient survival correlation [46]. Genes with log-rank *P*-value < 0.001 were deemed prognostic, where high expression correlated with low patient survival was an unfavourable prognosis, and increased survival was a favourable prognosis. In the MPeM interactome, we identified significant enrichment of genes that exhibited elevated expression, positively correlating with (i) unfavourable prognosis in liver, renal, pancreatic and lung cancers and (ii) favourable prognosis in testis, breast, thyroid and skin cancers (Supplementary Data File 6).

Next, we explored the relationship between interactome genes and other diseases using the DisGeNET database [47]. The top-5 diseases associated with MPeM were prostatic, mammary, stomach, liver and lung neoplasms, all at *P*-value < 1E-15 (Supplementary Data File 7). Notably, numerous novel interactors were linked to these diseases. For example, 13 novel interactors of MPeM-associated genes were associated with prostatic neoplasms (MPeM genes are shown in bold): **MET**-SLC26A4, **DPYD**-SULT2A1, **CTNNB1**-LAMB2, **IRS2**-MPO, **HRAS**-ZFP36L2, **VEGFB**-UCP3, **PRDM1**-HPGD, **NSD1**-NPR3, **KEAP1**-SLC5A5, **MET**-FOXA3, **RHEB**-NOS3, **HRAS**-HBG1 and **JAK1**-CBR1.

We then utilised Phenogrid from the MONARCH toolkit [48] to identify diseases phenotypically similar to MPeM. Phenogrid, an algorithm in the toolkit, determines shared phenotypes between two diseases. It gauges the information content of each phenotype (gene and disease associations) to quantify the observed similarity observed between the diseases. Ovarian fibroma (OF), desmoplastic small round cell tumour (DSRCT), Budd–Chiari syndrome (BCS) and primary peritoneal carcinoma (PPC) exhibited high phenotypic similarity to MPeM (similarity score > 80). We compiled 6, 43, 24, and 49 genes associated with OF, DSRCT, BCS, and PPC, respectively, and examined their enrichment in the MPeM interactome. Notably, significant enrichment was found for genes associated with DSRCT (*P*-value = 4.16E−04, odds ratio = 2.31) and PPC (*P*-value = 5.72E−08, odds ratio = 2.98).

Altogether, the gene enrichment patterns for diverse cancers uncovered from the interactome offer the potential to improve MPeM diagnosis and prognosis predictions, and customise treatment strategies.

### Interconnections to pleural mesothelioma interactome

**Fig. 4:**
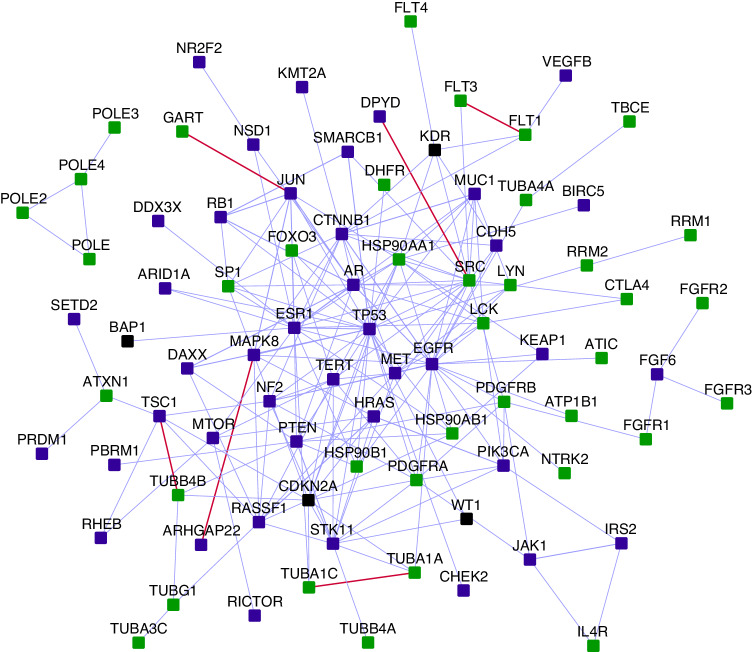
Interconnections of malignant peritoneal and pleural mesothelioma candidate genes: square-shaped blue nodes: malignant peritoneal mesothelioma (MPeM) candidates, square-shaped green nodes: malignant pleural mesothelioma (MPM) candidates, square-shaped black nodes: genes that are MPeM as well as MPM candidates. Light blue and red coloured edges indicate known and novel interactions respectively.

We sought to uncover the shared biological aspects between MPeM and malignant pleural mesothelioma (MPM). We compared the overlap of MPeM interactome with the MPM interactome [29], revealing 989 shared genes, a highly significant overlap (*P*-value = 3.18E−289, odds ratio = 2.92). This overlap included 4 core genes linked to both MPM and MPeM (BAP1, CDKN2A, KDR and WT1), 29 PPIs between MPM and MPeM core genes (one novel), and 21 novel interactors of MPM and MPeM core genes, alongside known interactors. Thirty-eight MPM-associated genes, 41 MPeM genes and the 4 genes common between them formed an intricately interconnected network of PPIs (Fig. 4). Six of these were novel PPIs (FLT1-FLT3, TUBA1A-TUBA1C, RHGAP22-MAPK8, DPYD-SRC, JUN-GART, and TSC1-TUBB4B).

Of the genes shared between the MPM and MPeM interactomes, 62.5% displayed differential expression in both MPeM and MPM transcriptomic datasets, i.e. in at least one of seven MPeM (Supplementary Data File 4) and one of six MPM datasets [29]. Notably, 33% of these differentially expressed genes were involved in the immune system (*P*-value < 1E−16). We identified a compact network (Supplementary Fig. 1) interconnecting 5 MPeM-associated and 5 MPM-associated genes via 19 known PPIs and 5 novel PPIs. This network highlighted the potential shared immune pathways that could contribute to tumour invasion and metastasis in both subtypes [49], notably IL-17 signalling via its modulator IKBKE [50] and T_H_17 cell differentiation via five genes, namely, MPeM-associated HSP90AA1 and HSP90AB1, MPM-associated JUN and MAPK8, and MPM-associated membrane protein MUC1 widely implicated in mesothelioma malignancy [51].

In summary, we uncovered a substantial shared gene pool between MPeM and MPM upon exploring their interactome overlaps. The majority of these genes showed altered expression in both MPeM and MPM transcriptomic datasets, predominantly in immune-related pathways. This underscores the significant role played by immune pathways in the progression of both mesothelioma subtypes, holding crucial implications for future research and therapeutic approaches.

### Potentially repurposable drugs for MPeM

We followed the established approach of comparing drug-induced versus disease-associated differential expression [52] to identify potential drugs for MPeM treatment. Utilising the BaseSpace Correlation software suite (https://www.nextbio.com) [41, 42], which previously helped identify repurposable drug candidates for schizophrenia [53] (currently undergoing clinical trials [54, 55]) and mesothelioma [29], we analysed pre-processed gene expression datasets.

We constructed the MPeM drug-protein interactome that shows the drugs that target any protein in the MPeM interactome. In total, 152 drugs (collected from Drug Bank [33]) were found to target 427 proteins, encompassing 16 MPeM-associated genes, 361 known interactors and 50 novel interactors. Our focus then turned to selecting 5 gene expression datasets pertinent to peritoneal mesothelioma. These included granulocytic myeloid-derived suppressor cells (G-MDSCs) sourced from spleens of mice with AB12 mesothelioma grafts versus naive neutrophils, as well as neutrophils infiltrating AB12 mesothelioma tumour grafts versus naive bone marrow-derived neutrophils (GSE43254 [56]). Additionally, datasets covering BCA-induced peritoneal mesothelioma versus non-transformed mesothelial cell line, O-NT-induced peritoneal mesothelioma versus non-transformed mesothelial cell line (GSE4682 [57]), and spontaneous malignant mesotheliomas from 2-year-old rats versus normal mesothelial Fred-PE cells (GSE47581 [58]) were included.

Then, we curated a list of chemical compounds with differential gene expression profiles (drug vs. no drug) that exhibited negative correlations with at least one of the five peritoneal mesothelioma differential gene expression datasets (disease vs. control). The rationale for choosing drugs that show a negative correlation with at least one of the five expression datasets is rooted in the complexity of the MPeM genetic landscape. This approach recognises the heterogeneous nature of MPeM and the inherent variability across its associated expression datasets. At the same time, it acknowledges the potential of drugs—even those that display correlation with only a single MPeM expression profile—to effectively target specific genes that might not exhibit uniform dysregulation across datasets.

**Fig. 5:**
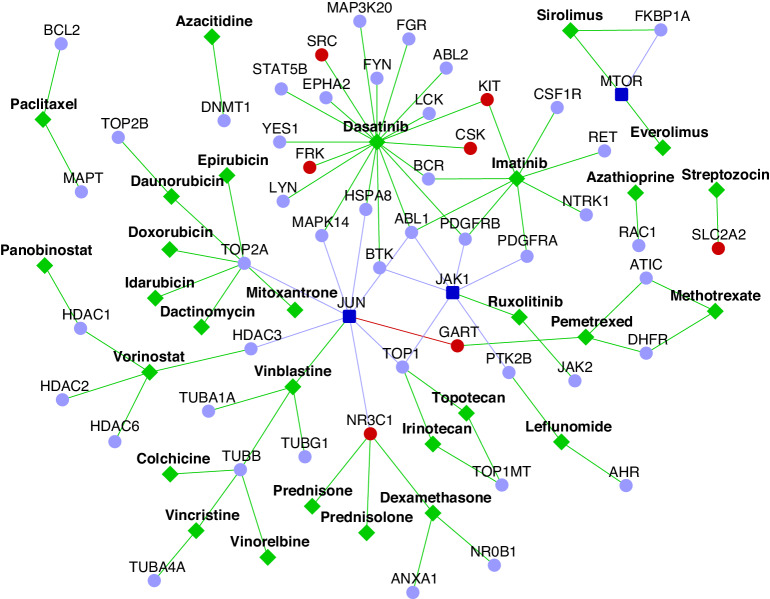
Repurposable drugs for malignant peritoneal mesothelioma (MPeM): the network shows 29 repurposable drugs (diamond-shaped green coloured nodes) that target the proteins in the MPeM interactome. MPeM candidates are shown as dark blue nodes, their known interactors are light blue and novel interactors are red.

Overall, we identified 39 drugs as potentially repurposable candidates for MPeM, including 23 that showed a negative correlation with two or more gene expression datasets and 16 negatively correlated with a single dataset (Supplementary Data Files 8–12). The literature review supported the biological validity of 29 (74%) out of these 39 drugs. These 29 drugs are shown in Fig. 5. Notably, 2 of these drugs (paclitaxel: NCT04000906 and imatinib: NCT00402766) are already in clinical trials for MPeM, and 2 others (pemetrexed and vinorelbine) are part of the standard therapy for mesothelioma [59]. In addition to this, the other shortlisted drugs exhibited activity relevant to MPeM (see Supplementary Note 1 for details). In short, irinotecan has exhibited effectiveness against peritoneal mesothelioma, pleural mesothelioma, and peritoneal metastasis. Clinical trials and tests in cell lines have demonstrated the efficacy of paclitaxel and sirolimus against peritoneal mesothelioma and peritoneal metastasis. Clinical trials, animal models, and cell lines have validated the efficacy of twelve drugs against malignant pleural mesothelioma, namely, epirubicin, panobinostat, doxorubicin, imatinib, vinblastine, idarubicin, azacitidine, vorinostat, dactinomycin, acetylcysteine, staurosporine, and quercetin. Six drugs have shown effectiveness against primary peritoneal cancer and peritoneal metastasis in other cancers, namely, ruxolitinib, daunorubicin, dasatinib, topotecan, dexamethasone, and nintedanib. Methotrexate, resveratrol, everolimus, and genistein have demonstrated efficacy against both malignant pleural mesothelioma and peritoneal metastasis or sclerosis. Mitoxantrone and vincristine have been proven effective in managing pleural/peritoneal effusions.

## Discussion

While multiple studies have examined MPeM genetics [5–13], this study employs the protein interactome to uncover the biological themes underlying the MPeM-associated genes. The MPeM interactome, constructed from over 4700 known and over 400 novel interactions of MPeM-associated genes from 9 studies, is examined using functional enrichment and transcriptome-based analyses to confirm its biological significance and to gain valuable insights into MPeM aetiology, as well as to identify potentially repurposable drugs. Although a study centred on a single biological hypothesis would have been advantageous, the absence of mechanistic research on MPeM compelled us to conduct an exploratory analysis, resulting in a comprehensive understanding of its functional landscape. The hypotheses generated herein can be explored further through in vitro and in vivo studies.

Given the limited biological information available for MPeM, integrating the MPeM interactome with transcriptomic evidence becomes crucial to distinguish true disease-associated genes from those unrelated to the disease, going beyond the core MPeM genes. We found that, within the interactome, over 75%—including more than 60% of novel interactors predicted for MPeM-associated genes—exhibited MPeM-related transcriptomic changes in humans and rodent models. Notably, 70% of these genes (1654 in total) had two or more pieces of supporting evidence. This integration of transcriptomic proof and the MPeM interactome effectively helped discern disease-associated genes from others. By overlaying disease-specific transcriptomic and genomic data onto the interactome, we could uncover an active sub-network of MPeM-associated genes (see Supplementary Data File 4) that possibly drives disease phenotypes [60]. The validity of our interactome-based approach is ascertained further by two factors: first, the unbiased identification of additional genes from the MPeM interactome, previously appearing in MPeM-related transcriptomic datasets, and second, their close functional proximity and interconnectedness with curated core genes harbouring MPeM-associated variants.

Biphasic MPeM, a rare subtype combining the common yet milder epithelioid type with the rarer, more severe sarcomatoid type, remains challenging to diagnose and manage due to limited reporting and occurrence [61, 62]. The enrichment of the interactome with genes unique to biphasic MPeM implies distinctive molecular mechanisms underlying this subtype, operating at the network level. The identification of new interactors specific to this subtype suggests unexplored pathways and potential treatment targets. The interactome—encompassing over 100 biphasic subtype genes—can be used as a resource for biomarker discovery and tailored therapies. This underscores the broader potential of interactome-based methods for uncovering complexities in rare cancer subtypes.

Differential diagnosis of MPeM is challenging due to its non-specific clinical symptoms and histological patterns, often leading to misdiagnosis of other cancers [63]. Three novel interactors of MPeM-associated genes—TACSTD2, IGF2, and SUSD2—can help differentiate OC/PPC from MPeM. Given that MPeM diagnosis currently relies on pathological evaluations [63], resources such as our interactome can be leveraged to develop immunohistochemical diagnostic panels, thereby improving diagnosis and treatment outcomes.

The interactome showed enrichment for spleen and thymus-specific genes. This corresponded to the identification of a hematopoiesis module in the MPeM interactome. Both spleen and thymus regulate extramedullary hematopoiesis, i.e. the production of blood cells outside the bone marrow, a phenomenon crucial for cancer progression, albeit less reported in solid tumours compared to myeloproliferative neoplasms [64]. When reported, the phenomenon often manifests as organomegaly (enlarged organ) [64]. Expansion of myeloid cells in the spleen through the process of extramedullary hematopoiesis resulting in splenomegaly has been observed in BAP1 (a core mesothelioma gene) knockout mice [65]. Although further investigations may be necessary to understand the functional implications, our finding suggests a potential link between extramedullary hematopoiesis and MPeM development.

The lack of enrichment of peritoneum-specific genes or genes specific to abdominal organs in the interactome is consistent with the absence of a distinct primary site for MPeM [66]. Pathological assessments commonly depict MPeM as diffusely spread throughout the abdominal cavity. However, additional research is necessary to determine if this lack of a primary site arises from the heterogeneity of MPeM-associated genes.

The modules and pathways identified from the interactome provide insights into processes spanning multiple biological levels that could contribute to the development and progression of peritoneal mesothelioma. Note that the majority of supporting evidence stems from pleural mesothelioma studies. Dysregulated covalent chromatin modification, including histone modifications and SUMOylation, can lead to genetic instability and epigenetic changes driving malignant transformation [67]. Altered mRNA metabolic and transcriptional processes might impact gene expression profiles [68, 69], while disrupted translation and post-translational events like deubiquitination could influence cellular proteomes [35, 70]. Furthermore, the activation of transmembrane receptor protein tyrosine kinase pathways, coupled with downstream MAPK cascades [71], and disrupted cell-cell junction assembly can enhance tumour cell survival and invasiveness [72], thereby promoting cancer progression.

The enrichment of cytokine signalling underscores the potential impact of inflammation, particularly the positive regulation of interleukin-6 (IL-6) production, on the progression of peritoneal mesothelioma. Indeed, elevated expression of an anti-apoptotic factor called survivin (BIRC5) induced by the cytokine IL-6 has been reported in MPeM patients; knockdown of this gene led to increased (spontaneous and drug-induced) apoptosis [12]. The IL-6 production module contained 13 novel interactors of MPeM core genes: **NR2F2**-SYCP3, **ESR1**-DDX43, **RB1**-LRCH1, **RB1**-PCDHB5, **MRE11**-GPR83, **PBRM1**-FBXW8, **RB1**-CDADC1, **MET**-FOXA3, **RB1**-CNTN3, **SMARCB1**-MYO18B, **TRIO**-DNAH5, **ARHGAP22**-ZNF488 and **SDHB** (and **MTOR**)-SLC45A1. Future studies could concentrate on examining these novel PPIs. This is particularly important because both chronic inflammation induced by abdominal surgeries and persistent peritoneal inflammation (i.e. chronic peritonitis) confer a risk of developing MPeM [1].

Lastly, among the MPeM core genes used for interactome construction, 56% (33 in total) were linked to chromosomal events such as copy number gain/loss, gene loss, deletion and gene rearrangement. Correspondingly, the interactome revealed enrichment for chromosome segregation as a functional module. Notably, we identified 7 novel PPIs that can be examined in experimental studies, with both the MPeM core genes and their novel interactors involved in chromosomal events (MPeM genes are shown in bold): **RASSF1**-LARS2, **ARID1B**-MTHFD1L, **RHEB**-CENPE, **VEGFB**-TFB1M, **JUN**-GART, **PTEN**-KIF20B and **KEAP1**-SENP1.

The pleural and peritoneal mesothelioma subtypes differ in their association with germline mutations, history of asbestos exposure, and post-operative complications, and have different median survival rates [2, 3]. Although several studies have examined the genomic features distinguishing them [73, 74], none have identified their underlying biological themes. We showed that more than 950 genes co-occurred commonly in MPM and MPeM interactomes, which is an approximately threefold higher enrichment of high statistical significance than expected. Moreover, this shared interactomic subspace that underlies the two distinct mesothelioma subtypes is likely driven by immune pathways. This observation is particularly relevant given the emerging potential of gene signatures from the mesothelioma tumour immune microenvironment to predict therapy responses [75].

Currently, the first-line chemotherapy regimen for MPeM involves pemetrexed/cisplatin, resulting in complete or partial responses in merely 26% of patients and disease stabilisation in only 45% of patients [1]. We identified several repurposable drugs for MPeM treatment, with over 70% showing effectiveness against peritoneal mesothelioma, pleural mesothelioma, peritoneal metastasis and/or primary peritoneal cancer in clinical trials, animal models or cell lines, confirming the credibility of the approach. However, the drug-associated expression profiles analysed in our study were induced in a wide variety of cell lines. Therefore, to advance clinical translation in MPeM, the effect of the shortlisted drugs should be examined in human peritoneal mesothelioma cell lines or animal models.

Overall, the study allows us to conceptualise MPeM as originating from disrupted interactions within the MPeM interactome due to genetic mutations or aberrant expression of MPeM-associated genes [76]. The disturbances capable of influencing this interactome can manifest across multiple levels. The genetic underpinnings of MPeM manifest across several organs outside of the peritoneum and abdominal organs, and are linked to processes operating at the genomic, transcriptomic, and proteomic levels. Perturbations within the immunological system also contribute to MPeM development, with immune-mediated pathways playing a critical role in the shared origins of pleural and peritoneal subtypes of mesothelioma. Additionally, MPeM shares genetic attributes with other malignancies, including (but not limited to) genes predictive of patient prognosis. It could be difficult to differentially diagnose some of these malignancies from MPeM upon phenotypic assessment. Overall, MPeM is a complex disorder warranting investigations from various perspectives.

Our study has a few limitations. For several analyses, we have used genetic data from animal models due to the absence of human patient data. Results from these should be interpreted with caution. Direct correlations of genes/proteins/phenotypes between animal models and humans require thorough characterisation in both species [77]. Also, our bioinformatics-based conclusions should be confirmed through experimental validation in pertinent tissues or cell lines.

In summary, our study provides a network-level view of MPeM-associated genes and their functional consequences. The MPeM interactome can serve as a functional landscape to integrate multi-omics data, informing genetic and biomedical studies seeking to improve clinical interventions in MPeM.

## Methods

### Compilation of MPeM-associated genes and prediction of novel interactions

A list of 59 MPeM-associated genes that harboured mutations, copy number aberrations, rearrangements or showed expression correlated with poor prognosis in MPeM patients or reduced cell survival or less favourable response to drugs in MPeM surgical specimens was compiled from eight studies [5–13]. Novel PPIs of the proteins encoded by these genes were predicted using the HiPPIP model that we developed [34]. Each MPeM protein (say N_1_) was paired with each of the other human proteins, say, (M_1_, M_2_,…M_n_), and each pair was evaluated with the HiPPIP model [34]. The predicted interactions of each of the MPeM proteins were extracted (namely, the pairs whose score is > 0.5, a threshold which — through computational evaluations and experimental validations — was revealed to indicate interacting partners with high confidence). The interactome figures were created using Cytoscape [78].

### Identification of functional modules

Functional gene modules were extracted using the HumanBase toolkit [43] (https://hb.flatironinstitute.org/). HumanBase uses shared k-nearest neighbours and the Louvain community-finding algorithm to cluster the genes sharing the same network neighbourhoods and similar GO biological processes into functional modules. The *p*-values of the terms enriched in the modules are calculated using Fisher’s exact test and the Benjamini–Hochberg method.

### Functional enrichment analysis

Biological process (Gene Ontology [79]), pathway (Reactome [80]) and disease (DisGeNET [47]) enrichments were computed using WebGestalt [44]. WebGestalt computes the distribution of genes belonging to a particular functional category in the input list and compares it with the background distribution of genes belonging to this functional category among all the genes that belong to any functional category in the database selected by the user. The statistical significance of functional category enrichment is computed using Fisher’s exact test and corrected using the Benjamini-Hochberg method for multiple test adjustment. Annotations with FDR-corrected *p*-value < 0.05 were considered significant.

### Tissue-specific expression analysis

Tissue-specificity of the genes in the MPeM interactome was checked using TissueEnrich [81]. The analysis was based on tissue-specific genes compiled from GTEx and Mouse ENCODE [38, 39]. This included ‘tissue-enriched genes’ with at least 5-folds higher mRNA levels in a particular tissue compared to all the other tissues, ‘group-enriched genes’ with at least 5-folds higher mRNA levels in a group of 2–7 tissues and ‘tissue-enhanced genes’ with at least 5-folds higher mRNA levels in a particular tissue compared to average levels in all tissues.

### Network overlap analysis

Statistical significance of the overlaps between genes in the MPeM and MPeM interactomes was computed based on hypergeometric distribution.

### Identification of prognostic cancer genes

Data for the correlation of gene expression and a fraction of the patient population surviving after treatment of 20 cancer types was taken from Pathology Atlas [46]. Genes with log-rank *P*-value < 0.001 were considered to be prognostic. Unfavourable prognosis indicates a positive correlation of high gene expression with reduced patient survival.

### Identification of repurposable drugs

The list of chemical compounds whose gene expression profiles correlated negatively with 5 gene expression datasets associated with peritoneal mesothelioma was compiled using the BaseSpace correlation software (https://www.nextbio.com) (List 1). The datasets considered were granulocytic myeloid-derived suppressor cells (G-MDSCs) from spleens of mice bearing AB12 mesothelioma grafts versus naive neutrophils, neutrophils infiltrating AB12 mesothelioma tumour grafts versus naive bone marrow-derived neutrophils (GSE43254 [56]), BCA induced peritoneal mesothelioma versus non-transformed mesothelial cell line, O-NT induced peritoneal mesothelioma versus non-transformed mesothelial cell line (GSE4682 [57]) and spontaneous malignant mesotheliomas from 2-year-old rats versus normal mesothelial Fred-PE cells (GSE47581 [58]). Next, we identified drugs that targeted at least one gene in in the MPeM interactome using Drug Bank [33]. We then compared List 1 and List 2 to identify the drugs that not only target proteins in the interactome but are also negatively correlated with MPeM-associated gene expression profiles.

## Supplementary information

Supplementary-Data-Files

Supplementary Information

## Data Availability

The MPeM core genes used for interactome construction, the complete list of PPIs in the MPeM interactome and the list of novel interactors in the interactome have been made available as Supplementary Data File 1, Supplementary Data File 2 and Supplementary Data File 3, respectively.
