# Supplementary material for: Malignant peritoneal mesothelioma interactome with 417 novel protein–protein interactions
Source: BJC Rep. 2024 May 24;2:42. doi: 10.1038/s44276-024-00062-w (PMC11524009; doi:10.1038/s44276-024-00062-w)
Supplement: Supplementary file 2 — Supplementary Information [file 44276_2024_62_MOESM2_ESM.docx]

## Supplementary Information

Malignant Peritoneal Mesothelioma Interactome with 417 Novel Protein-Protein Interactions

**Authors:**

Kalyani B. Karunakaran^1^ and Madhavi K. Ganapathiraju^2^*

**Affiliations:**

^1^Supercomputer Education and Research Centre, Indian Institute of Science, Bengaluru, 560012, India

^2^Department of Biomedical Informatics, School of Medicine,

Intelligent Systems Program, School of Computing and Information,

University of Pittsburgh, Pittsburgh, PA, USA

***Correspondence to:** Madhavi K. Ganapathiraju ([madhavi@pitt.edu](mailto:madhavi@pitt.edu))

This file contains:

- Supplementary Note 1
- Supplementary Figure 1
- Supplementary References 1 to 53

**Supplementary Note 1**

**Literature evidence supporting the clinical validity of repurposable drugs**

One drug has shown activity against peritoneal mesothelioma, pleural mesothelioma and peritoneal metastasis (irinotecan). Although ineffective as a single agent,^1^ **irinotecan** elicited modest response rates and showed an acceptable toxicity profile in malignant pleural mesothelioma clinical trials, and produced an inhibitory effect on mesothelioma cell lines in combination with a p53 activator.^2-4^ **Irinotecan** was shown to be effective and tolerable in gastric cancer patients with peritoneal seeding.^5^ Importantly, **irinotecan** in combination with cisplatin showed efficacy and tolerability against peritoneal mesothelioma in a clinical setting.^6^

Two drugs have shown activity against peritoneal mesothelioma in clinical settings and peritoneal metastasis in clinical trials/cell lines (paclitaxel and sirolimus). **Paclitaxel** appeared to be ineffective against MPM both as a single agent as well as in combination with other agents such as filgrastim and cisplatin.^7-9^ However, it has led to the complete remission of an MPeM patient for 20 months,^10^ and clinical efficacy and an acceptable toxicity profile in ovarian, pancreatic and gastric cancers with peritoneal metastasis or serosal exposure.^11,12^ **Sirolimus** inhibited proliferation and increased cell death in MPM cell lines in combination with cisplatin, inhibited epithelial-to-mesenchymal transition in peritoneal mesothelial cell lines, and showed clinical efficacy in a patient with benign multicystic peritoneal mesothelioma.^13-15^

Twelve drugs have shown activity against malignant pleural mesothelioma in clinical trials, animal models or cell lines (epirubicin, panobinostat, doxorubicin, imatinib, vinblastine, idarubicin, azacitidine, vorinostat, dactinomycin, acetylcysteine, staurosporine and quercetin). **Epirubicin** has shown a modest 10-20% response rate in malignant mesothelioma patients.^16^ **Panobinostat** has been shown to have an inhibitory effect in mesothelioma cell lines and tumors in murine xenograft models.^17^ **Doxorubicin** has shown tolerable toxicity and improvement in the quality of life of MPM patients.^18^ **Imatinib** has shown both cytotoxicity and apoptosis in PDGFRB-positive mesothelioma cell lines, and has shown modest clinical efficacy in malignant mesothelioma patients.^19,20^ **Vinblastine** has shown improved progression-free survival rate and acceptable toxicity in combination with methotrexate and platinum.^21-23^ Both **idarubicin** and **dactinomycin** showed more cytotoxicity in MPM cell lines compared with pemetrexed and cisplatin.^24^ **Azacitidine** has shown inhibitory activity against malignant mesothelioma in clinical trials.^25^ **Vorinostat** induced apoptosis in mesothelioma cell lines.^26^ However, it did not provide any therapeutic benefit in patients with pleural mesothelioma.^27^ **Acetylcysteine** and **quercetin** have shown dose-dependent inhibition and time- and dose-dependent inhibition in malignant mesothelioma cell lines respectively.^28-30^ **Staurosporine** was effective against mesothelioma tumors in murine xenograft models.^31^

Six drugs have shown activity against primary peritoneal cancer or peritoneal metastasis in other cancers (ruxolitinib, daunorubicin, dasatinib, topotecan, dexamethasone and nintedanib). The gene expression profile induced by **ruxolitinib** was shown to be negatively correlated with all the 5 peritoneal mesothelioma datasets. **Ruxolitinib** in combination with paclitaxel has been shown to inhibit tumor growth in a mouse model of advanced ovarian cancer with peritoneal metastasis.^32^ **Daunorubicin** induced side effects and showed no clinical activity against MPM.^33^ However, treatment with **daunorubicin** led to complete remission of a gastric Kaposi’s sarcoma patient with peritoneal metastasis.^34^ **Dasatinib** showed inhibitory activity against peritoneal metastasis in a murine xenograft model of gastric cancer.^35^ **Topotecan** demonstrated clinical efficacy and tolerability in primary peritoneal carcinoma.^36^ **Dexamethasone** protected peritoneal mesothelial cells from epithelial-to-mesenchymal transition by acting on MAPK, GSK-3β and SNAI1.^37^ **Nintedanib** has been shown to inhibit peritoneal fibrosis in a mouse model by blocking mesothelial-to-mesenchymal transition.^38^

Four drugs have shown activity both against malignant pleural mesothelioma and peritoneal metastasis or sclerosis (methotrexate, resveratrol, everolimus and genistein). **Methotrexate** was shown to be clinically active against MPM in combination with gemcitabine, and against peritoneal metastasis in advanced gastric cancer.^21,39-41^ **Everolimus** induced AMPK/p38-mediated apoptosis in MPM cell lines and showed clinical activity in encapsulating peritoneal sclerosis.^42-44^ **Resveratrol** has produced an inhibitory effect on mesothelioma cell lines as a single agent as well as in combination with clofarabine, and has inhibited the adhesion of ovarian cancer cells to peritoneal mesothelial cells in vitro.^45-48^ **Genisten** has shown inhibitory activity against matrix metalloproteinases in malignant mesothelioma cell lines, and against peritoneal metastasis in intestinal adenocarcinomas in Wistar rats.^49,50^

Two drugs have been shown to be effective in pleural/peritoneal effusions (mitoxantrone and vincristine). **Mitoxantrone** has shown only modest clinical activity in malignant mesothelioma.^51^ However, this drug has shown activity against pleural effusions in cancer patients.^52^ **Vincristine** was active against malignant peritoneal effusions in a murine xenograft model of Ehrlich ascites carcinoma.^53^

**
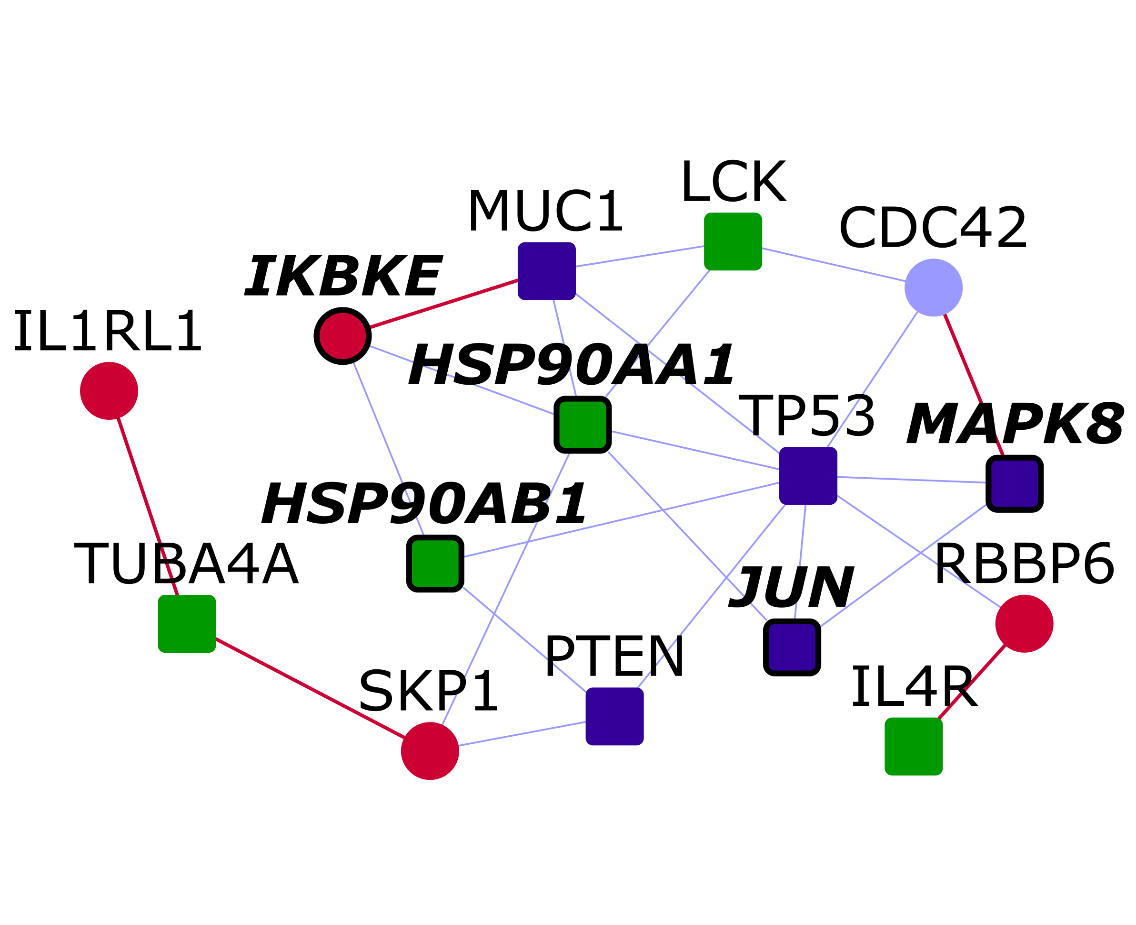
Supplementary Figure 1: IL-17 signaling deregulation as a mechanism underlying MPM and MPeM.** Square-shaped blue nodes: malignant peritoneal mesothelioma (MPeM) candidates, square-shaped green nodes: malignant pleural mesothelioma (MPM) candidates, genes with black border and bold italicized labels: genes that are that are involved in IL-17 signaling and T_H_17 differentiation pathways. Light blue and red colored nodes/edges indicate known and novel interactors/interactions respectively.

**Supplementary References**

1 Kindler, H. L. *et al.* Irinotecan for malignant mesothelioma: a phase II trial by the Cancer and Leukemia Group B. *Lung Cancer* **48**, 423-428 (2005).

2 Fennell, D. A. *et al.* Efficacy and safety of first‐or second‐line irinotecan, cisplatin, and mitomycin in mesothelioma. *Cancer* **109**, 93-99 (2007).

3 Han, B. *et al.* Combined use of irinotecan and p53 activator enhances growth inhibition of mesothelioma cells. *FEBS Open bio* **10**, 2375-2387 (2020).

4 Koda, Y. *et al.* Irinotecan and Gemcitabine as Second-Line Treatment in Patients with Malignant Pleural Mesothelioma following Platinum plus Pemetrexed Chemotherapy: A Retrospective Study. *Oncology*, 1-8 (2020).

5 Choi, M. K. *et al.* Phase I study of intraperitoneal irinotecan in patients with gastric adenocarcinoma with peritoneal seeding. *Cancer chemotherapy and pharmacology* **67**, 5-11 (2011).

6 Le, D. T., Deavers, M., Hunt, K., Malpica, A. & Verschraegen, C. F. Cisplatin and irinotecan (CPT-11) for peritoneal mesothelioma. *Cancer investigation* **21**, 682-689 (2003).

7 Van Meerbeeck, J. *et al.* Paclitaxel for malignant pleural mesothelioma: a phase II study of the EORTC Lung Cancer Cooperative Group. *British journal of cancer* **74**, 961-963 (1996).

8 Vogelzang, N. *et al.* High-dose paclitaxel plus G-CSF for malignant mesothelioma: CALGB phase II study 9234. *Annals of Oncology* **10**, 597-600 (1999).

9 Fizazi, K. *et al.* Combination raltitrexed (Tomudex®)–oxaliplatin: a step forward in the struggle against mesothelioma? The Institut Gustave Roussy experience with chemotherapy and chemo-immunotherapy in mesothelioma. *European Journal of Cancer* **36**, 1514-1521 (2000).

10 Bednar, M. & Chahinian, P. in *Ann Meet Am Soc Clin Oncol.* 496a.

11 Yamada, S. *et al.* Phase I/II study of adding intraperitoneal paclitaxel in patients with pancreatic cancer and peritoneal metastasis. *The British journal of surgery* **107**, 1811 (2020).

12 Kitayama, J., Ishigami, H., Yamaguchi, H., Emoto, S. & Watanabe, T. Intraperitoneal paclitaxel is useful as adjuvant chemotherapy for advanced gastric cancer with serosal exposure. *Case Reports in Oncology* **7**, 58-64 (2014).

13 Hartman, M.-L., Esposito, J. M., Yeap, B. Y. & Sugarbaker, D. J. Combined treatment with cisplatin and sirolimus to enhance cell death in human mesothelioma. *The Journal of thoracic and cardiovascular surgery* **139**, 1233-1240 (2010).

14 Stallone, G., Infante, B., Cormio, L., Macarini, L. & Grandaliano, G. Rapamycin treatment for benign multicystic peritoneal mesothelioma: a rare disease with a difficult management. *The American Journal of Case Reports* **18**, 627 (2017).

15 Xiang, S. *et al.* Rapamycin inhibits epithelial‐to‐mesenchymal transition of peritoneal mesothelium cells through regulation of Rho GTPases. *The FEBS journal* **283**, 2309-2325 (2016).

16 Lee, J. J., Trizna, Z., Hsu, T., Spitz, M. R. & Hong, W. K. A statistical analysis of the reliability and classification error in application of the mutagen sensitivity assay. *Cancer Epidemiology and Prevention Biomarkers* **5**, 191-197 (1996).

17 Gultekin, K. *et al.* Effects of cisplatin and panobinostat on human mesothelial (Met-5A) and malignant pleural mesothelioma (MSTO-211H) cells. *Genet Mol Res* **12**, 5405-5413 (2013).

18 Arrieta, O. *et al.* First-line chemotherapy with liposomal doxorubicin plus cisplatin for patients with advanced malignant pleural mesothelioma: phase II trial. *British journal of cancer* **106**, 1027-1032 (2012).

19 Bertino, P. *et al.* Preliminary data suggestive of a novel translational approach to mesothelioma treatment: imatinib mesylate with gemcitabine or pemetrexed. *Thorax* **62**, 690-695 (2007).

20 Tsao, A. S. *et al.* Phase I trial of cisplatin, pemetrexed, and imatinib mesylate in chemonaive patients with unresectable malignant pleural mesothelioma. *Clinical lung cancer* **15**, 197-201 (2014).

21 Hunt, K. J., Longton, G., Williams, M. A. & Livingston, R. B. Treatment of malignant mesothelioma with methotrexate and vinblastine, with or without platinum chemotherapy. *Chest* **109**, 1239-1242 (1996).

22 Tsavaris, N. *et al.* Combination chemotherapy with cisplatin-vinblastine in malignant mesothelioma. *Lung Cancer* **11**, 299-303 (1994).

23 Muers, M. *et al.* BTS randomised feasibility study of active symptom control with or without chemotherapy in malignant pleural mesothelioma: ISRCTN 54469112. *Thorax* **59**, 144-148 (2004).

24 Kanellakis, N. I. *et al.* Patient-derived malignant pleural mesothelioma cell cultures: a tool to advance biomarker-driven treatments. *Thorax* **75**, 1004-1008 (2020).

25 Vogelzang, N. J. *et al.* Dihydro‐5‐azacytidine in malignant mesothelioma: A Phase II trial demonstrating activity accompanied by cardiac toxicity. *Cancer: Interdisciplinary International Journal of the American Cancer Society* **79**, 2237-2242 (1997).

26 Hurwitz, J. L. *et al.* Vorinostat/SAHA-induced apoptosis in malignant mesothelioma is FLIP/caspase 8-dependent and HR23B-independent. *European Journal of Cancer* **48**, 1096-1107 (2012).

27 Krug, L. M. *et al.* Vorinostat in patients with advanced malignant pleural mesothelioma who have progressed on previous chemotherapy (VANTAGE-014): a phase 3, double-blind, randomised, placebo-controlled trial. *The Lancet Oncology* **16**, 447-456 (2015).

28 Tanaka, M. *et al.* Inhibition of NADPH oxidase 4 induces apoptosis in malignant mesothelioma: Role of reactive oxygen species. *Oncology reports* **34**, 1726-1732 (2015).

29 Demiroglu-Zergeroglu, A., Basara-Cigerim, B., Kilic, E. & Yanikkaya-Demirel, G. The investigation of effects of quercetin and its combination with cisplatin on malignant mesothelioma cells in vitro. *BioMed Research International* **2010** (2010).

30 Lee, Y.-J. *et al.* Quercetin exerts preferential cytotoxic effects on malignant mesothelioma cells by inducing p53 expression, caspase-3 activation, and apoptosis. *Molecular & Cellular Toxicology* **11**, 295-305 (2015).

31 Kinoh, H. *et al.* Nanomedicines eradicating cancer stem-like cells in vivo by pH-triggered intracellular cooperative action of loaded drugs. *ACS nano* **10**, 5643-5655 (2016).

32 Han, E. S. *et al.* Ruxolitinib synergistically enhances the anti-tumor activity of paclitaxel in human ovarian cancer. *Oncotarget* **9**, 24304 (2018).

33 Steele, J. *et al.* Phase II trial of liposomal daunorubicin in malignant pleural mesothelioma. *Annals of oncology* **12**, 497-499 (2001).

34 Fléchon, A., Lombard-Bohas, C., Boulez, J., Blay, J.-Y. & Scoazec, J.-Y. Complete response of an HIV negative gastric Kaposi’s sarcoma (KS) patient with peritoneal carcinomatosis by liposomal daunorubicin treatment. *Annals of oncology* **12**, 275-276 (2001).

35 Kurashige, J. *et al.* Integrated molecular profiling of human gastric cancer identifies DDR2 as a potential regulator of peritoneal dissemination. *Scientific reports* **6**, 22371 (2016).

36 Safra, T. *et al.* Weekly topotecan for recurrent ovarian, fallopian tube and primary peritoneal carcinoma: Tolerability and efficacy study—The Israeli experience. *International Journal of Gynecologic Cancer* **23** (2013).

37 Jang, Y.-H. *et al.* Effects of dexamethasone on the TGF-β 1-induced epithelial-to-mesenchymal transition in human peritoneal mesothelial cells. *Laboratory investigation* **93**, 194-206 (2013).

38 Liu, F. *et al.* Nintedanib inhibits the development and progression of peritoneal fibrosis. (2020).

39 Kuribayashi, K. *et al.* Methotrexate and gemcitabine combination chemotherapy for the treatment of malignant pleural mesothelioma. *Molecular and clinical oncology* **1**, 639-642 (2013).

40 Solheim, Ø., Saeter, G., Finnanger, A. & Stenwig, A. High-dose methotrexate in the treatment of malignant mesothelioma of the pleura. A phase II study. *British journal of cancer* **65**, 956-960 (1992).

41 Yamao, T. *et al.* Phase II study of sequential methotrexate and 5-fluorouracil chemotherapy against peritoneally disseminated gastric cancer with malignant ascites: a report from the Gastrointestinal Oncology Study Group of the Japan Clinical Oncology Group, JCOG 9603 Trial. *Japanese journal of clinical oncology* **34**, 316-322 (2004).

42 Ou, S.-H. I. *et al.* SWOG S0722: phase II study of mTOR inhibitor everolimus (RAD001) in advanced malignant pleural mesothelioma (MPM). *Journal of Thoracic Oncology* **10**, 387-391 (2015).

43 Pignochino, Y. *et al.* The combination of sorafenib and everolimus shows antitumor activity in preclinical models of malignant pleural mesothelioma. *BMC cancer* **15**, 1-13 (2015).

44 Duman, S. *et al.* in *Advances in peritoneal dialysis. Conference on Peritoneal Dialysis.* 104-110.

45 Batirel, S. *et al.* Resveratrol Inhibits Malign Pleural Mesothelioma Cell Proliferation Through Antioxidant System. *Free Radical Biology and Medicine* **86**, S33-S34 (2015).

46 Lee, Y.-J. *et al.* Resveratrol contributes to chemosensitivity of malignant mesothelioma cells with activation of p53. *Food and chemical toxicology* **63**, 153-160 (2014).

47 Ganapathiraju, M. K. *et al.* Schizophrenia interactome with 504 novel protein–protein interactions. *npj Schizophrenia* **2**, 16012 (2016).

48 Mikuła-Pietrasik, J., Sosińska, P. & Książek, K. Resveratrol inhibits ovarian cancer cell adhesion to peritoneal mesothelium in vitro by modulating the production of α5β1 integrins and hyaluronic acid. *Gynecologic oncology* **134**, 624-630 (2014).

49 Liu, Z. & Klominek, J. Regulation of matrix metalloprotease activity in malignant mesothelioma cell lines by growth factors. *Thorax* **58**, 198-203 (2003).

50 Iishi, H. *et al.* Genistein attenuates peritoneal metastasis of azoxymethane‐induced intestinal adenocarcinomas in Wistar rats. *International journal of cancer* **86**, 416-420 (2000).

51 van Breukelen, F. J. *et al.* Mitoxantrone in malignant pleural mesothelioma: a study by the EORTC Lung Cancer Cooperative Group. *European Journal of Cancer and Clinical Oncology* **27**, 1627-1629 (1991).

52 Maiche, A., Virkkunen, P., Kontkanen, T., Möykkynen, K. & Porkka, K. Bleomycin and mitoxantrone in the treatment of malignant pleural effusions. A comparative study. *American journal of clinical oncology* **16**, 50-53 (1993).

53 Bairy, K. *et al.* Evaluation of intraperitoneal vincristine in malignant peritoneal effusion. *Indian journal of physiology and pharmacology* **47**, 270-278 (2003).
